# Rapid mitochondrial genome sequencing based on Oxford Nanopore Sequencing and a proxy for vertebrate species identification

**DOI:** 10.1002/ece3.6151

**Published:** 2020-03-11

**Authors:** Nicolás D. Franco‐Sierra, Juan F. Díaz‐Nieto

**Affiliations:** ^1^ Grupo de investigación en Biodiversidad, Evolución y Conservación (BEC) Departamento de Ciencias Biológicas, Escuela de Ciencias Universidad EAFIT Medellín Colombia

**Keywords:** genome skimming, mitochondrial DNA sequencing, MinION, molecular species identification, vertebrate species identification

## Abstract

Molecular information is crucial for species identification when facing challenging morphology‐based specimen identifications. The use of DNA barcodes partially solves this problem, but in some cases when PCR is not an option (i.e., primers are not available, problems in reaction standardization), amplification‐free approaches could be an optimal alternative. Recent advances in DNA sequencing, like the MinION device from Oxford Nanopore Technologies (ONT), allow to obtain genomic data with low laboratory and technical requirements, and at a relatively low cost. In this study, we explore ONT sequencing for molecular species identification from a total DNA sample obtained from a neotropical rodent and we also test the technology for complete mitochondrial genome reconstruction via genome skimming. We were able to obtain “de novo” the complete mitogenome of a specimen from the genus *Melanomys* (Cricetidae: Sigmodontinae) with average depth coverage of 78X using ONT‐only data and by combining multiple assembly routines. Our pipeline for an automated species identification was able to identify the sample using unassembled sequence data (raw) in a reasonable computing time, which was substantially reduced when a priori information related to the organism identity was known. Our findings suggest ONT sequencing as a suitable candidate to solve species identification problems in metazoan nonmodel organisms and generate complete mtDNA datasets.

## Graphical abstract

The findings of this study suggest ONT sequencing as a suitable candidate to solve species identification problems in metazoan nonmodel organisms and generate complete mtDNA datasets.

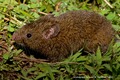

## 1 INTRODUCTION

Species are the fundamental study unit in biology so that accurate species identification is a vital process in the biological and medical sciences (Mayr, 1982; de Queiroz, 2005). DNA sequencing has been increasingly used in recent years for species identification given the challenges associated with morphological identification (e.g., need of adult individuals, specimens preserved in optimal conditions, inability for recognition of cryptic species, among many others; Avise, 1994; Crawford et al., 2013; Mendoza et al., 2016). Yet, traditional DNA‐based identification requires processing samples in a laboratory, using DNA sequencing techniques, which traditionally involve robust equipment, methods that preclude obtaining sequences of nonmodel organisms (e.g., specific primers for PCR sequencing), and high‐cost reagents that are not available at many laboratories (Erlich, 2015; Sboner, Mu, Greenbaum, Auerbach, & Gerstein, 2011; Wetterstrand, 2018).

Recent developments in DNA sequencing techniques, such as single‐molecule sequencing detection like the nanopore‐based method from Oxford Nanopore Technologies (ONT), are a breakthrough in molecular biology due to its multiple advantages such as long sequencing reads, portability (pocket‐sized device called MinION), reduced cost, and relative simplicity for its setup and operation compared with the traditional sequencing platforms (Jain, Olsen, Paten, & Akeson, 2016; Laver et al., 2015). Given those benefits, a new range of applications can be explored in fields such as microbiology, human genetics, basic genome research, microbiome studies, and clinical and animal research (Norris, Workman, Fan, Eshleman, & Timp, 2016; Schmidt et al., 2017). Caveats in this sequencing method are mostly associated with its high error rate (~10%), which is primarily attributed to the basecalling process (Magi, Giusti, & Tattini, 2016). Consequently, computational advances on basecalling from the raw signal are still required to obtain higher sequence read quality compared with short‐read technologies, some misreadings can be captured from modified nucleotides and homopolymer sequences, and in consequence, indels can be introduced (Magi et al., 2016). Despite of this, the use of the technology has been demonstrated with outstanding success in genomic surveillance of Ebola and Zika virus outbreaks (Faria et al., 2016; Quick et al., 2016, 2017), improved reconstruction in genomes of well‐studied model organisms such as Gram‐negative bacterium *Escherichia coli* (Quick, Quinlan, & Loman, 2014), yeast *Saccharomyces cerevisiae* (Salazar et al., 2017), and nematode *Caenorhabditis elegans* (Tyson et al., 2018), and even the resolution of complicated genomic regions (telomeres, centromeres, HLA locus, Y chromosome) of the human genome (Jain, Koren, et al., 2018; Jain, Olsen, et al., 2018).

In the field of DNA‐based species identification, ONT sequencing has been widely used in bacterial and viral applications, especially for clinical research, environmental, and microbiome studies (Benítez‐Páez, Portune, & Sanz, 2016; Greninger et al., 2015). As a consequence, most computational pipelines and efforts have been developed for those taxa (model organisms), in tools such as WIMP (What's in my pot?; Juul et al., 2015) and Kraken (Wood & Salzberg, 2014).

Regarding molecular identification of metazoan organisms, advances have been done using barcodes in studies performed for identification of amphibians and reptiles in the Ecuadorian rainforest (Pomerantz et al., 2018), and cost‐effective methods benchmarked with samples of hundreds of dipteran and hymenopteran insects (Srivathsan et al., 2018). However, these approaches rely on the use of DNA barcoding, and therefore, they require amplification by PCR, implying additional laboratory steps and reagents in order to obtain the desired sequence. In addition, when working with nonmodel organisms or with less‐studied biotic groups at the molecular level, adequate primers are not available to achieve a successful amplification reaction (Schäffer, Zachos, & Koblmüller, 2017).

DNA barcodes have been proved as a cost‐effective, accessible, and reliable solution to the species identification problem (Hebert, Cywinska, Ball, & DeWaard, 2003). Since its proposal, many data of DNA barcodes have been obtained and released, especially for mitochondrial *COI* barcode (cytochrome *c* oxidase subunit I) in metazoans resolving a wide range of questions (taxonomic, evolutionary, and identification) in many insect, avian, and other animal clades (Blagoev et al., 2016; Hebert, Ratnasingham, & Waard, 2003; Kerr, Lijtmaer, Barreira, Hebert, & Tubaro, 2009; Stoeckle, 2003). Nonetheless, some taxonomic groups would benefit from high‐quality reference sequence sets at genomic and mitogenomic scales (Lessa, Cook, D'Elía, & Opazo, 2014), especially to solve discrepancies observed in single gene‐based analysis (e.g., gene tree–species tree discordance in molecular systematics). Highly diverse taxa usually lack sufficient molecular information to properly address most of these questions. A particular example of such diverse group can be found in the sigmodontine rodents (e.g., cotton and rice rats, grass mice), a subfamily of the Neotropical family Cricetidae consisting of 86 extant genera and about 400 species, of which 85 genera and 381 species occur in South America, inhabiting all the available ecoregions of the continent (D'Elía & Pardiñas 2015). To date, although a notable proportion of species within the subfamily have available DNA sequences, these are mostly associated with the mitochondrial Cytochrome *b* oxidase gene (*CYTB)* and only four species (<1% of the sigmodontine diversity) have their complete mitochondrial genomes sequenced (4 records on GenBank accessed 4 September 2019), a dramatically low number for the genomic era (Coissac, Hollingsworth, Lavergne, & Taberlet, 2016). Sure enough, more extensive taxon sampling is required to address phylogenomic approaches in this group (Lessa et al., 2014).

The cost–benefit and accessibility of ONT allow obtaining genome‐wide and organellar DNA sequences in situ without the need (and avoid the associated problems) of PCR‐based amplification. Previous efforts have been made to obtain mitochondrial genomes using ONT sequencing data, but to overcome the read noisiness and improve assembly quality, short‐read data from Illumina platform were also included (Chandler et al., 2017; Torres et al., 2018). Herein, we propose that with enough mitochondrial DNA coverage (genome skimming), the mitogenome could be accurately reconstructed using ONT‐only data combining several computational assembly routines to solve known issues and error‐prone sites in the sequence. Although noisiness will still be present in sequence reads, this information is valuable as a rapid alternative for species identification purposes. We explore the sequencing use of the MinION from ONT as a promising tool for mitogenomics and molecular species identification of nonmodel organisms with a PCR‐free approach. As a case of study, we present the first complete mitochondrial genome assembled using ONT‐only data of the South American sigmodontine rodent of the genus *Melanomys* and evaluate an assembly‐free method for its molecular identification.

## 2 MATERIALS AND METHODS

### 2.1 DNA extraction

**FIGURE 1:**
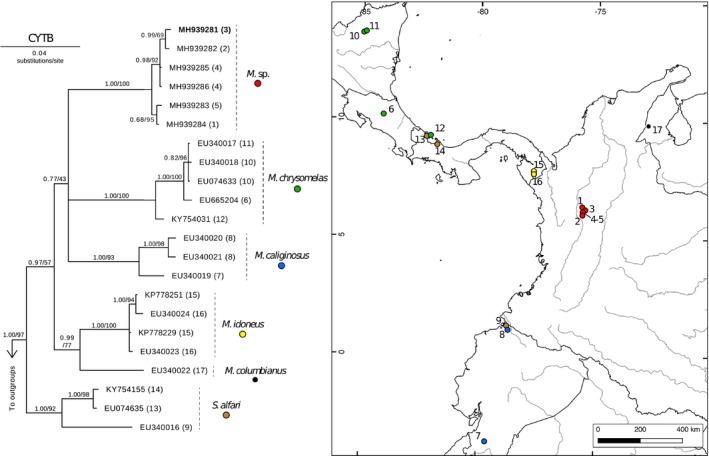
Left. Bayesian inference phylogenetic reconstruction of *Melanomys* and *Sigmodontomys CYTB* sequences. Terminals include GenBank accession numbers followed by their corresponding locality number (in parenthesis). Bolded terminal corresponds to sample JFD1322. Support values are shown on nodes (posterior probabilities/Bootstrap support). Right. Geographic distribution of material sequenced for our phylogenetic analyses

The aim of this work was to evaluate the use of ONT sequencing as a tool for molecular species identification of nonmodel organisms. To achieve this, fieldwork was conducted at a periurban locality at municipality of Envigado, Antioquia, Colombia (Figure 1), and we used Sherman traps to capture any rodent of the speciose subfamily Sigmodontinae. A small rodent identified in the field as *Melanomys caliginosus* was captured (collector number JFD1322) and deposited at the biological collection of Universidad EAFIT (Medellín, Colombia). The whole liver of the animal was extracted, and a fraction was used fresh for DNA extraction. The remaining liver was stored frozen at −80°C.

Two different methods were used for whole DNA extraction: (a) GenElute™ Mammalian Genomic DNA Miniprep Kit Protocol (Sigma‐Aldrich) following manufacturer's instructions, and (b) a DNA extraction protocol designed to obtain high molecular weight DNA (Quick, 2018). Both protocols used 25 mg of fresh liver as start material. For the high molecular weight DNA (HMW) protocol, the following procedure was used: The liver tissue (25 mg) was resuspended by gentle pipette mixing in 200 μl PBS (buffer phosphate saline 1×). 10 ml TLB was added (100 mM NaCl, 10 mM Tris‐Cl, pH 8.0, 25 mM EDTA, pH 8.0, 0.5% (w/v) SDS, 20 μg/ml Sigma RNase A solution), vortexed at full speed for 5 s, and incubated at 37°C for 1 hr. 50 μl Proteinase K (Sigma) was added and mixed by slow end‐over‐end rotation three times followed by 2 hr at 50°C with gentle mixing every 30 min. The lysate was phenol‐purified by adding 10 ml TE saturated phenol on phase‐lock gel falcon tubes (Dow Corning vacuum grease). Tubes were incubated at room temperature and rotated at 20 rpm for 10 min to achieve a homogeneous emulsion. The emulsion was centrifuged at 2846 *g* for 10 min in order to achieve complete phase separation. Aqueous phase was retained, and an additional phenol:chloroform (1:1) purification step was performed under the same conditions. The DNA was precipitated by the addition of 4 ml 5 M ammonium acetate and 30 ml ice‐cold absolute ethanol. DNA was pelleted by centrifugation at 2,846 *g* for 10 min and recovered with a glass hook followed by washing twice in 70% ethanol. After spinning down at 10,000 g, ethanol was removed followed by 15 min of drying at room temperature. 100 μl EB (elution buffer: 10 mM Tris‐Cl, pH 8.0, 0.2% Triton X‐100) was added to the DNA and left at 5°C during 48 hr to fully resuspend. DNA integrity was assessed by agarose gel electrophoresis. DNA extracted from both methods was visually inspected on 0.5% agarose gel in 1× TAE buffer (40 mM Tris–acetate and 1.0 mM EDTA, pH 8.3) for 14 hr at 30 V to ensure good separation of HMW fragments if present.

### 2.2 Oxford Nanopore Sequencing run

MinION device was used for total DNA sequencing using Ligation Sequencing Kit 1D—SQK‐LSK108. Two sequencing experiments were developed: (a) the sample processed using DNA extraction kit and (b) the one processed using HMW protocol. The sequencing library preparation procedure was the same for both experiments and was performed as follows: ~3 μg of total DNA (measured by fluorometry using Qubit 3.0 dsDNA BR assay) was adjusted to a volume of 45 μl with nuclease‐free water (NFW). End repair and dA‐tailing were performed on extracted DNA using NEBNext Ultra II End Repair/dA‐Tailing Module (NEB E7546S) by adding 7 μl of reaction buffer, 3 μl of enzyme mix, and 5 μl of NFW. This mixture was incubated at 20°C for 5 min and 65°C for 5 min. Then, a 1× volume (60 μl) AMPure XP clean‐up was performed and the DNA was eluted in 31 μl NFW. 1 μl aliquot was quantified by Qubit to ensure retention of at least 700 ng DNA. Adapter ligation was performed by adding 20 μl Adaptor Mix (SQK‐LSK108: AMX1D) and 50 μl NEB Blunt/TA Master Mix (NEB M0367S) to the 30 μl end‐prepped DNA, mixing gently by flicking, and incubating at room temperature for 30 min. The adaptor‐ligated DNA was cleaned up by adding a 0.4× volume (40 μl) of AMPure XP beads, incubating for 5 min at room temperature and resuspending the pellet twice in 140 μl ABB buffer (SQK‐LSK108: ABB). The purified DNA was resuspended by adding 15 μl of elution buffer (SQK‐LSK108: ELB), resuspending the beads, incubating at room temperature for 10 min, pelleting the beads again, and transferring the supernatant (presequencing mix) to a new tube. 1 μl aliquot was quantified by Qubit to ensure a retention of at least 430 ng DNA.

Sequencing was performed using MinION R9 flow cells: FLO‐MIN106 (R9.4 chemistry) for the (a) sample treated using commercial GenElute DNA extraction kit, and FLO‐MIN107 (R9.5 chemistry) for the (b) sample processed using HMW‐DNA protocol. Flow cells were primed prior sequencing by loading 800 μl of priming mix (48% v/v running fuel buffer (SQK‐LSK108:RBF) in NFW) into the flow cell via the priming port, waiting for 5 min, lifting the SpotON sample port cover, and loading 200 μl of priming mix via the priming port, always avoiding the introduction of any air bubbles. Sequencing library, of both runs, was prepared by adding 35 μl RBF, 25.5 Library Loading Beads (Library Loading Bead Kit EXP‐LLB001:LLB), and 0.5 NFW to 14 μl of the presequencing mix. The library was loaded dropwise onto the SpotON sample port using a P100 tip set to 75 μl and entered the flow cell by capillary action.

MinION sequencing run was controlled using MinKNOW software (version 1.4.2) setting a runtime of 48 hr for each experiment without live basecalling. Subsequently, the raw signal for each run (stored as FAST5 files) was basecalled using ONT Albacore Sequencing Pipeline Software (version 2.1.10) in order to obtain sequencing reads in FASTQ and FAST5 (including sequence data) formats. In order to assess the performance of both sequencing runs, base and read counts were performed, and read length histograms for each dataset and for comparing runs were plotted. Those tasks were performed using NanoStat 1.1.0, NanoComp 0.16.4, and NanoPlot 1.13.0, which are tools from NanoPack (De Coster, D'Hert, Schultz, Cruts, & Broeckhoven, 2018).

### 2.3 ONT sequencing data analysis and mitogenome reconstruction

Accurate species identification relies on available data deposited in public databases to perform comparisons. For vertebrates, it is common to find abundant mitochondrial DNA sequences, mostly from *COI* and *CYTB* genes since they are widely used as DNA barcodes (Hebert, Cywinska, et al., 2003). Consequently, because we are interested in evaluating the usefulness of the MinION for rapid species identification, mitochondrial DNA was selected as our target locus for this study. Reads presumably assigned to mtDNA were selected by a BLAST‐based strategy using mtBlaster (a script written by our group for this task https://github.com/nidafra92/squirrel-project/blob/master/mtblaster.py). *Sigmodon hispidus* mitogenome (GenBank: KY707311.1) was used as reference sequence for this search since it was the closest evolutionary‐related organism to the study specimen with an available complete mitogenome. mtBlaster takes as input DNA sequencing reads in FASTQ file format and aligns them to a reference mitochondrial genome, and it retrieves a FASTQ file containing the subset of reads that matched to the reference.

During the early stages of the mitogenome reconstruction, some genes were difficult to assemble as they showed premature stop codons, indicating misassembly. In an effort to resolve this issue, we produced four different assemblies (using reads from the sequencing experiment of HMW‐DNA sample since its theoretical depth was above 30×) varying parameters that aimed to correct Nanopore typical basecalling errors (i.e., homopolymers and modified nucleotides). Detailed workflow for each assembly is as follows: (assemblies 1–3 were performed using selected reads after Albacore basecalling) *Assembly 1*. Draft assembly of mtDNA reads was performed using MiniMap 2.1/Miniasm 1 (Li, 2016, 2018). Draft assembly was polished applying 5 rounds of mapping reads against to the previous assembly using BWA 0.7.12‐r1039 (Li & Durbin, 2010) and subsequently polishing with RACON 1.2.1 (Vaser, Sović, Nagarajan, & Šikić, 2017). A final polishing step was implemented with Nanopolish 0.8.5 (Loman, Quick, & Simpson, 2015) on the 5 times of RACON‐polished assembly. For this final polishing, the following parameters were modified in the “Nanopolish variants” command line: Ploidy status was set to “1” (‐‐ploidy 1), the maximum haplotype combinations were restricted to 1 (‐x 1), and the homopolymer caller was enabled (‐‐fix‐homopolymers). *Assembly 2*. Draft assembly of mtDNA reads with Minimap2/Miniasm was polished 5 times by RACON, and a final polish was performed with Nanopolish enabling methylation aware feature (‐q dcm) and disabling homopolymer calling correction. *Assembly 3*. Draft assembly of mtDNA reads with Minimap2/Miniasm was polished 5 times by RACON and polished twice with Nanopolish enabling methylation aware feature (‐q dcm) and using homopolymer calling correction. *Assembly 4*. A draft assembly was performed using mtDNA reads with Phred score higher than Q7 but called via Chiron 0.3 deep learning‐based basecaller (Teng et al., 2018). Those reads were assembled using Canu v1.7 (Koren et al., 2017) (genomeSize = 16.3k ‐nanopore‐raw chiron_mtDNAreads.fasta correctedErrorRate = 0.2 minOverlapLength = 250 corMhapSensitivity = high corMinCoverage = 0 contigFilter = “2 0 1.0 0.5 0”), polished twice using Racon and 3 times by Nanopolish (‐‐max‐rounds 750) using both homopolymer caller and methylation aware (‐q dcm) features.

**FIGURE 2:**
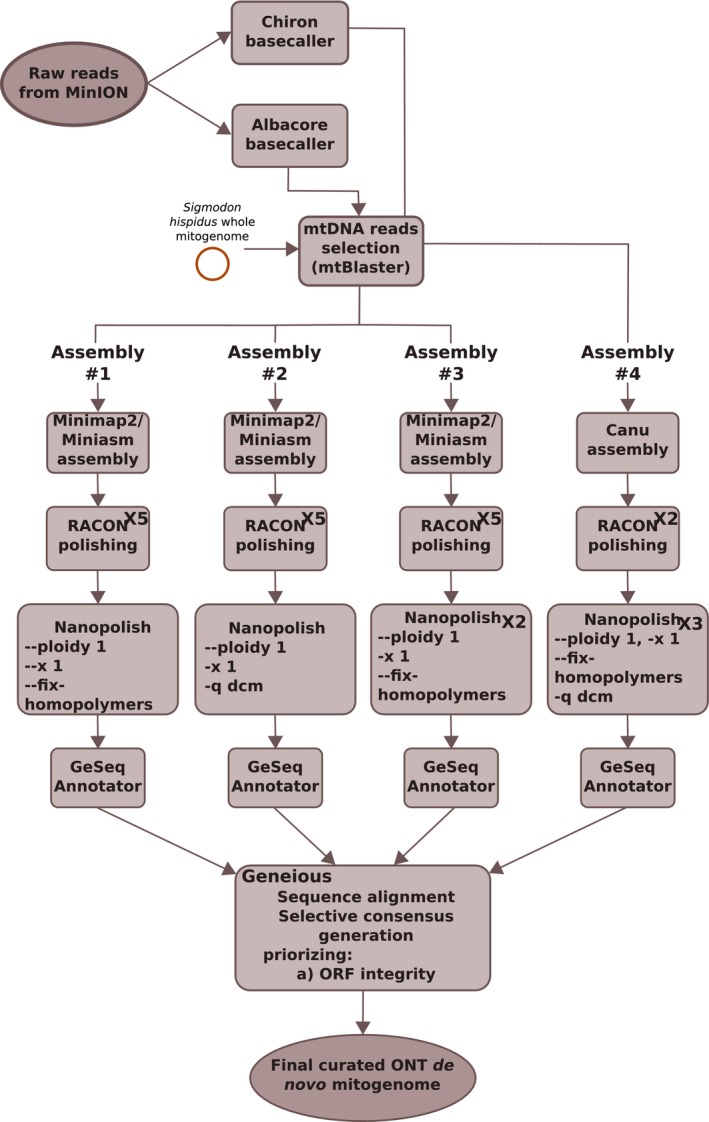
Computational pipeline implemented for the mitochondrial genome reconstruction based on ONT‐only sequencing data

The reconstructed mitogenome sequences were annotated using GeSeq (Annotation of Organellar Genomes) from CHLOROBOX web server hosted at the Max Planck Institute of Molecular Plant Physiology (Tillich et al., 2017) modifying the following parameters: “circular sequence” option was checked, sequence source was set to “Mitochondrial,” tRNAscan‐SE v2.0 in “Mammalia Mitochondrial tRNAs” mode was enabled, and Server References from NCBI were selected including all RefSeqs under Muroidea taxonomic rank (NCBI:txid337687). Finally, the four annotated mitogenome assemblies were aligned in Geneious R11.1.4 (http://www.geneious.com, Kearse et al., 2012) using Geneious aligner algorithm. The fully aligned region was inspected, and a consensus was produced carefully checking for polymorphic positions to ensure the integrity of the reading frames of the protein‐coding genes and the expected secondary structure of tRNAs and rRNAs. The complete procedure for the mitogenome reconstruction is schematized in Figure 2. Coverage depth of the final assembly was assessed through mapping all the mtDNA reads to the final consensus sequence after all the polishing, correction, and curation steps using BWA‐mem. Finally, Tablet 1.17 (Milne et al., 2013) was used for visualization and inspection of the generated alignment.

### 2.4 Confirming ONT sequence accuracy using Sanger sequencing

Error rates in ONT sequencing data are relatively high compared with those from short‐read sequencing approaches, and difficulties in assemblies have been observed in A‐T‐biased sequences, homopolymer regions, and modified nucleotides (e.g., DNA methylation), all these features present in mtDNA. To confirm the accuracy of our assembled mitogenome, two regions from mtDNA were amplified by PCR and then sequenced by Sanger method to analyze possible discrepancies with respect to our ONT‐generated sequences. We therefore obtained sequences of the Cytochrome *b* oxidase gene (*CYTB*) and Cytochrome *c* oxidase subunit I gene (*COI*) using primers reported in Percequillo, Weksler, & Costa (2011) and Ivanova, Clare, and Borisenko (2012), respectively. For both loci, PCR amplification was implemented in 25 μl reactions using the initially extracted DNA as template, Taq DNA polymerase recombinant (Thermo Scientific), and following recommended concentrations of primers, dNTPs, buffer, and MgCl2. For the *CYTB*, thermal cycling conditions included 2 min of initial denaturation at 95°C, followed by 5 cycles × (30 s 95°C, 40 s 52°C, 1 min 72°C), 5 cycles × (30 s 95°C, 40 s 54°C, 1 min 72°C), 5 cycles × (30 s 95°C, 40 s 56°C, 1 min 72°C), 25 cycles × (30 s 95°C, 40 s 57°C, 1 min 72°C), and a final 10‐min extension at 72°C. For *COI*, thermal cycling conditions varied slightly with 2 min at 95°C, 35 cycles × (30 s 95°C, 40 s 52°C, 1 min 72°C), and 10 min at 72°C. All the obtained amplicons were sequenced using amplification primers and dye‐terminator chemistry on an ABI‐3730xl automated sequencer. Sanger sequences for each marker were edited, assembled, and examined in Geneious R11.1.4 (http://www.geneious.com, Kearse et al., 2012). The obtained consensus sequences (after assembling forward and reverse reads) for each locus (*CYTB*, *COI*) were aligned in Geneious to the complete mitogenome assembly generated with ONT data.

### 2.5 Species identification using mitogenomic ONT nucleotides

To perform a molecular identification based on ONT raw data, the following considerations were taken. Most molecular species identification is based on DNA barcode methods (Hebert, Cywinska, et al., 2003). For almost all animal groups, a segment of the mitochondrial Cytochrome *c* oxidase subunit I gene (known as “*COX1*” or “*COI*”) has been sequenced. In addition to this marker, other mitochondrial regions (depending on the taxonomic group) are used, such as *CYTB*, *COX2*, *NAD1*, *NAD2,* or mitochondrial coded rRNAs (Feldman & Omland, 2005; Goebel, Donnelly, & Atz, 1999; Vences, Thomas, Meijden, Chiari, & Vieites, 2005); consequently, public data from mitochondrial loci are available for a wide range of animal groups. Given its high mutation rates, uniparental heritability, and relatively fast coalescent times (Hudson & Turelli, 2003), mtDNA sequences have been widely used for molecular identification of metazoan organisms (Blagoev et al., 2016; Hebert, Ratnasingham, et al., 2003; Kerr et al., 2009; Stoeckle, 2003). Therefore, our molecular identification strategy from unassembled ONT reads is mtDNA‐based.

Basecalled reads from HMW‐DNA were initially prefiltered to obtain only putative metazoan mtDNA reads. In order to do this, first, a metazoan reference database was constructed using complete mitogenome sequences of well‐known model organisms: the nematode *Caenorhabditis elegans* (GenBank: NC_001328.1), fruit fly *Drosophila melanogaster* (GenBank: NC_024511.2), mosquito *Anopheles gambiae* (GenBank: NC_002084.1), chicken *Gallus gallus* (GenBank: NC_001323.1), tunicate *Ciona intestinalis* (GenBank: NC_017929.1), laboratory mouse *Mus musculus* (GenBank: NC_005089.1), laboratory rat *Rattus norvegicus* (GenBank: AY172581.1), human *Homo sapiens* (GenBank: NC_012920.1), zebrafish *Danio rerio* (GenBank: NC_002333.2), pufferfish *Takifugu rubripes* (GenBank: NC_004299.1), and African clawed frog *Xenopus laevis* (GenBank: NC_001573.1). Second, an initial nucleotide BLAST (blastn) run was performed, using BLAST+ 2.8 (Camacho et al., 2009) with 60% identity cutoff value of our ONT raw reads against the metazoan model organism mtDNA database. The low identity cutoff was established taking into account the average ONT error rate of ~10% and 75% identity threshold to safeguard homology at the nucleotide level. Positive hits from this search were classified as presumably metazoan mtDNA reads.

For the species identification, the recovered mtDNA reads were blasted against a new custom database (different to the metazoan model organism mtDNA database) containing 4,686,865 metazoan mtDNA sequences from GenBank (accessed on 11 August 2018). For this search, BLAST parameters were set as follows: max_target_seqs = 1, word_size = 11, gapopen = 2, gapextend = 2, penalty=−3, reward = 2, max_hsps = 1, perc_identity = 85, and task = blastn. Stringency of BLAST parameters was lowered to mitigate the effect of mismatch and indels in positive reads due to read noisiness inherent to the sequencing technology. We computed a weighted frequency score for each read based on BLAST percentage of identity. For instance, read #1 matched to a DNA sequence of species A with 95% identity and read #2 matched to species B with 90% identity; at this point, candidate species are species A (0.95 score) and species B (0.90). Those scores were updated as more reads were identified using the database and this criterion. This information was summarized and represented in pie charts to inform the identification of the organism from whose DNA was used in the sequencing run.

The identification process (above described) was performed as three independent analyses, reducing the size of the queried database (at three different taxonomic levels) to evaluate performance and runtimes of the routine when a priori information of the sample identity was available: (a) when no information is available (the complete metazoan mtDNA dataset), (b) when sample identity is known at order level, and (c) when sample identity is known at family level. All BLAST searches were executed in parallel in a computer cluster at the Apolo Scientific Computing Center (Universidad EAFIT) using DC‐BLAST (Divide and Conquer BLAST for HPC) utility (Yim & Cushman, 2017). The setup used for executions consisted of two HPC nodes with 32 cores per node, 64 GB RAM per node, Intel^®^ Xeon^®^ CPU E5‐2683 v4 @ 2.10 GHz processor.

### 2.6 Phylogenetic reconstruction

Rapid molecular identification methods, as the one proposed here, can be used as a proxy to species identification; nonetheless, results need to be taken cautiously because they mostly rely on the taxonomic identification of the sequences uploaded to public databases, and the gene/taxon sampling found in such repositories. Phylogenetic reconstruction (using molecular data, morphological characters, or both) can be an important method for species identification when dense taxon sampling is available (for some examples, see: Davalos & Jansa, 2004; Díaz‐Nieto, Jansa, & Voss, 2016a; Díaz‐Nieto, Jansa, & Voss, 2016b; Jansa & Weksler, 2004; Weksler, 2006); consequently, and in order to test the results of a rapid and automated species identification procedure (see Section 2.5) we developed an independent species identification study using phylogenetic methods, including an expanded taxon sampling. For this purpose, our gene sampling was restricted to the *CYTB* gene, an appropriate marker because it has a denser taxon and geographic sampling available for sigmodontine rodents. Although the genus *Melanomys* lacks a recent revision, for our taxon sampling we follow the proposal of Hanson and Bradley (2008) and Weksler and Lóss (2015) who recognize 6 species in the genus: *M. caliginosus*, *M. chrysomelas*, *M. columbianus*, *M. idoneus*, *M. robustulus*, and *M. zunigae*. Our ingroup included all sequences available in GenBank for the genus (which corresponds to all the recognized species with the exception of *M. robustulus* and *M. zunigae*)*,* in addition to our JFD1322 *CYTB* sequence and 5 other *CYTB* sequences from *Melanomys* specimens collected in the north of Cordillera Central. Also, because the genus *Melanomys* has been previously recognized as paraphyletic by some authors (Hanson & Bradley, 2008; Pine, Timm, & Weksler, 2012; Weksler, 2006) our ingroup included GenBank sequences from the genus *Sigmodontomys*. Finally, we used sequences from the species *Oryzomys palustris* and *Tanyuromys aphrastus* as outgroups. Our complete taxon sampling can be found in Table S1.

DNA sequences were aligned using MUSCLE 7.271 (Katoh & Standley, 2013) in ‐‐auto mode. The best‐fitting nucleotide substitution model was determined by the BIC in jModelTest 2.1.10 (Darriba, Taboada, Doallo, & Posada, 2012). Our *CYTB* matrix was analyzed using Bayesian inference (BI) and maximum likelihood (ML) searches. Bayesian inference was implemented in MrBayes v3.2 (Ronquist et al., 2012) by running 4 independent Markov chain Monte Carlo (MCMC) analyses for 5,000,000 generations each, sampling every 500 generations, including 1 cold chain and 3 heated chains, and implementing the optimal substitution model from jModelTest. A maximum clade credibility consensus tree was generated from the sampled trees after discarding a relative burn‐in fraction of 10% using TreeAnnotator v2.5.2 (Bouckaert et al., 2014). Maximum likelihood analysis was implemented in Garli 2.01 (Zwickl, 2006) based on 4 independent searches, using the same substitution model from jModelTest, and evaluating nodal support by running 1,000 bootstrap pseudoreplicated datasets. Bipartitions of the bootstrap searches were summarized on the best ML topology using SumTrees from Dendropy package (Sukumaran & Holder, 2010). Finally, we calculated both uncorrected (*p*‐distance) and corrected (Tamura–Nei and Gamma) *CYTB* distances between and within clades using MEGA X (Kumar, Stecher, Li, Knyaz, & Tamura, 2018). All DNA distance calculations were made considering all substitutions (transitions + transversions) and pairwise deletion treatment for gap/missing data.

## 3 RESULTS

### 3.1 Performance of ONT sequencing runs

**FIGURE 3:**
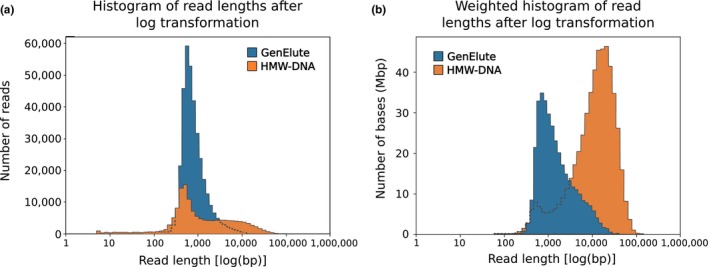
Distribution of sequencing yield generated from both libraries (DNA Extraction Kit and HMW‐DNA). (a) Distribution of read length after log transformation for both sequencing libraries. (b) Weighted histogram of read length after log transformation for both sequencing libraries

Two successful sequencing runs were obtained from the DNA recovered using both extraction protocols. A higher DNA yield was obtained from the HMW‐DNA extraction protocol (65.4 µg) compared with that obtained using the DNA extraction kit (4 µg). DNA integrity assessment on agarose gel showed little fragmentation for the sample treated with the HMW‐DNA extraction protocol, whereas a highly smeared band was observed for the sample treated with the DNA extraction kit, suggesting considerable fragmentation of the DNA (Figure S1). Accordingly, higher sequencing yield was observed for the sample obtained from the HMW‐DNA extraction protocol (526.18 Mbp) compared with the yield obtained from the DNA extraction kit (354.37 Mbp). Fewer reads were generated from the HMW‐DNA library (139,431) compared with the extraction kit library (352,088) as average read length was higher for the former (3,773 bp) and lower for the latter (1,006.5 bp). Details on DNA characteristics and sequencing statistics for both sequencing libraries can be observed in Table 1. Figure 3 shows read length distributions of both libraries with longer fragments sequenced in the HMW‐DNA run compared with the DNA extraction kit run.

**TABLE 1:** Sequencing performance of two MinION runs using total DNA extracted from the liver of specimen JFD1322 of *Melanomys* sp

	DNA extraction method	
	GenElute extraction kit	HMW‐DNA protocol
DNA yield (ng DNA/25 mg tissue)	4,077	65,400
Flow cell version	FLO‐MIN106 (R9.4)	FLO‐MIN107 (R9.5)
Sequencing run yield (Mbp)	354.37	526.18
Number of reads	352,088	139,431
Average read length (bp)	1,006.5	3,773
Average base quality (Phred score)	8.9	6.4
Longest read (bp)	36,744	149,424

After filtering to obtain the highest possible amount of mitochondrial DNA reads (mtDNA reads) from our target sample (JFD1322) using the metazoan reference database, low mtDNA yield was obtained from each library: 0.36% from the DNA extraction kit library and 0.53% from the HMW‐DNA library. Next, we decided to apply a second filter based on read length by only keeping those reads longer than 6 kbp in order to decrease the possibility of capturing NUMTs (nuclear mitochondrial segments). After this second filter, we retained 24 reads (avg. length of 8,862 bp) from the DNA extraction kit library and 127 reads (avg. length of 11,026 bp) from the HMW‐DNA library. Assuming a 16‐kpb‐long mitochondrial genome, the theoretical mtDNA sequencing depth was 13X for the DNA extraction kit library and 88X for the HMW‐DNA library. Consequently, only the HMW‐DNA reads could be successfully assembled (depth > 30×) into a mitochondrial genome. Full statistics on mtDNA reads can be seen in Table 2.

**TABLE 2:** Sequencing yield of mitochondrial DNA for both runs inferred from the total DNA sequencing data using mtBlaster

	DNA extraction method	
	GenElute	HMW‐DNA protocol
mtDNA sequencing yield (Mbp)	1.28 (0.36%)	2.8 (0.53%)
Number of mtDNA reads	1,045	1,609
Average mtDNA read length (bp)	1,227	1,756
Average base quality (Phred score)	9.4	8.1
Median mtDNA read length (bp)	744	919
mtDNA in reads > 6 kbp (Mbp)	0.212 (0.06%)	1.4 (0.27%)
Number of mtDNA reads > 6 kbp	24	127
Average mtDNA reads > 6 kbp read length (bp)	8,862	11,026
Theoretical mtDNA sequencing depth (for a 16 kbp mitogenome)	13.25×	87.5×

### 3.2 Complete mitochondrial genome of *Melanomys*

**FIGURE 4:**
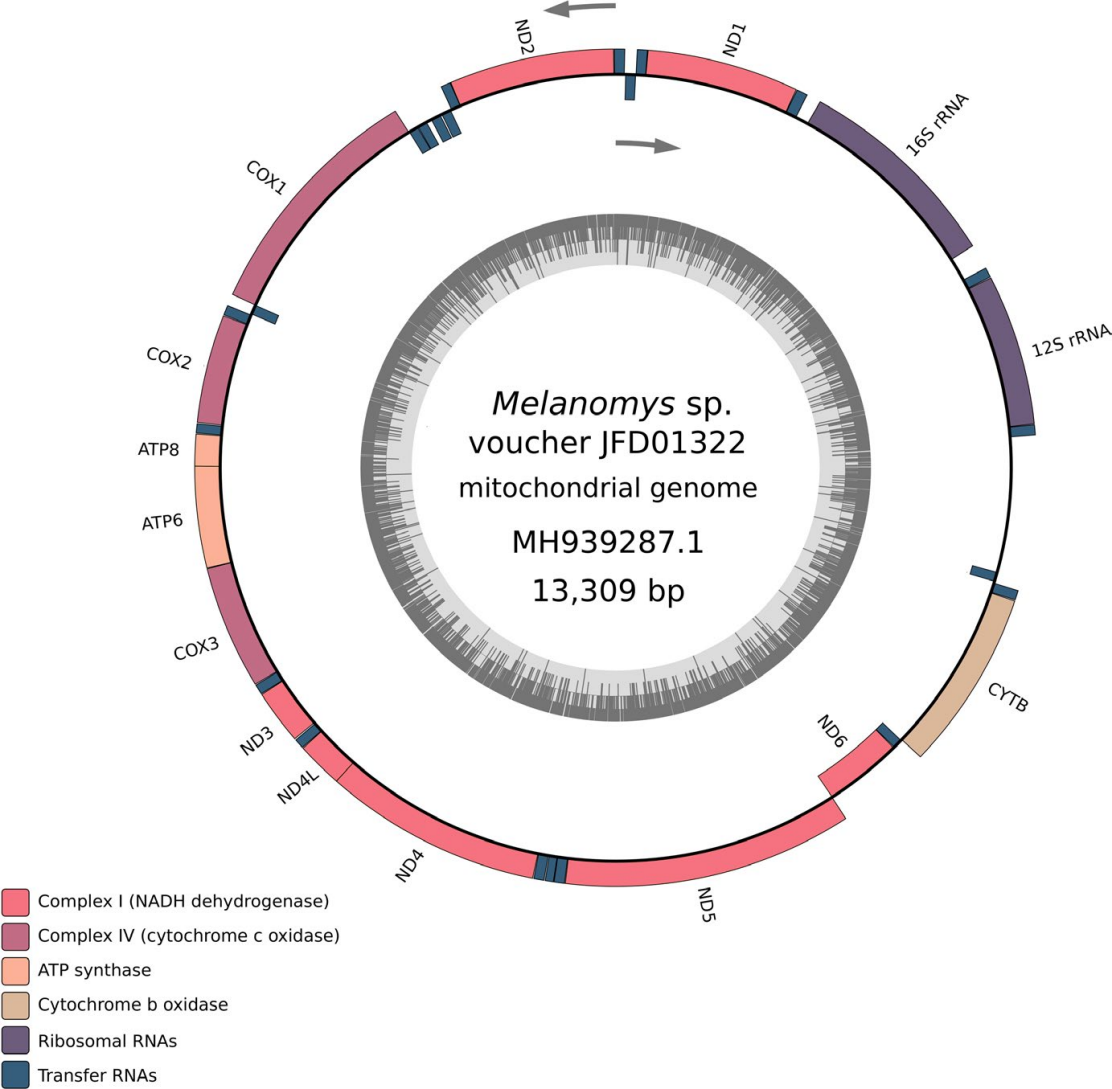
Circular map of the mitochondrial genome of *Melanomys* sp. (specimen JFD1322) obtained from ONT sequencing. Color legend indicating gene type is shown in bottom left, inner circle shows %GC content, and light gray arrows indicate direction of transcription

A mtDNA molecule was successfully reconstructed from the four assembly strategies derived from the HMW‐DNA library reads with an average coverage depth of 77.5× (max depth of 84×). As a result from our DNA‐sequenced ONT‐only data, we obtained the resulting mitogenome assembly of *Melanomys* specimen JFD1322 as a closed circular 16,309‐bp molecule (GenBank accession number MH939287), which contains the typical set of 37 mitochondrial genes (13 protein‐coding genes, 22 tRNAs, and two rRNAs; Figure 4). A total of 28 genes were transcribed on the heavy‐coding strand, while the rest (9) were transcribed on the light‐coding strand. The nucleotide composition of the entire mitogenome (A: 35.0%, T: 28.5%, C: 24.9%, and G: 11.6%) is A + T‐biased (63.5%) and exhibits positive AT‐skew (0.102) and negative GC‐skew (−0.362) values. Coding sequences occupy 91.7% of the total genome length. The protein‐coding genes encompassed 11,387 bp of the entire assembled sequence (69.8%). Accuracy of the obtained assembly from ONT data was assessed through confirmation with Sanger sequencing of partial sequences of *COI* (662 bp) and *CYTB* (1,122 bp) gene. The segments of *COI* (GenBank: MH939280) and *CYTB* (GenBank: MH939281) genes that were PCR‐amplified and Sanger sequenced were identical (100% identity) to the final curated consensus mitogenome obtained from ONT data.

### 3.3 Species‐level identification

**FIGURE 5:**
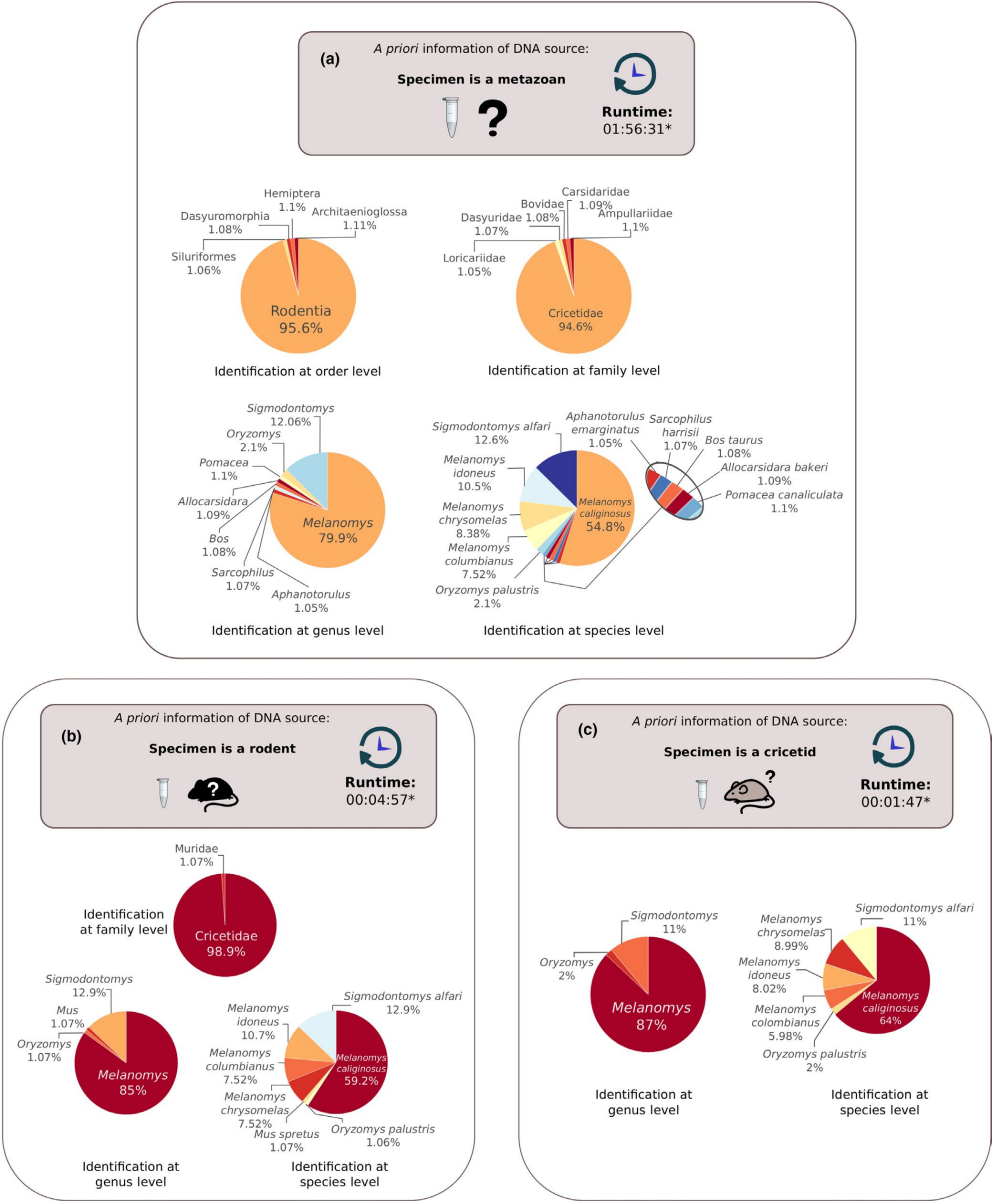
Species identification using ONT nucleotide raw data. (a) Results when previous information of the sample identity is limited to Metazoa. (b) Results when previous information of sample identity is available at order level (Rodentia). (c) Results when previous information of sample identity is available at family level (Cricetidae). * See text for hardware details

The taxonomic identity of our sample (JFD1322) was obtained using the complete dataset from the HMW‐DNA library using our BLAST‐based methodology. We observed considerable differences in running time among the three identification analyses; for instance, when the search was performed without any prior information on the sample identification using the whole mtDNA database of Metazoa, the analysis took 1:56:31 hr in our computer cluster, whereas when performed against our order‐level (Rodentia) dataset, the analysis took 00:04:57 min, and when the analysis was performed using the family‐level (Cricetidae) dataset, it took 00:01:47 min. Results of the three analyses showed comparable identifications, with ~80% of the reads associated with the genus *Melanomys*, and the highest proportion of reads (54.8%–64.0%) matched to the species *Melanomys caliginosus* (Figure 5). Noteworthy, when the identification search was performed with the complete metazoan dataset, few hits (5.39%) showed no specificity with any rodent but with other animal clades such as snails (Architaenioglossa), psyllids (Hemiptera), and catfish (Siluriformes; Figure 5a). All three mentioned taxa are commonly found in streams of Colombian montane forests (Stevenson, Pérez‐Torres, & Muñoz‐Saba, 2006), which is the habitat where the *Melanomys* specimen sequenced in this study was captured. A graphical summary of these results can be seen in Figure 5.

### 3.4 Phylogenetic reconstruction

Our *CYTB* dataset includes 22 ingroup sequences (19 sequences from the genus *Melanomys* and 3 from the genus *Sigmodontomys*) and 3 outgroup sequences (1 from the genus *Tanyuromys* and 2 from the species *Oryzomys palustris*) ranging in length between 801 and 1,143 bp for a total nucleotide coverage of 96.70%. Opposed to several recent phylogenetic analyses of sigmodontine rodents where the genus *Melanomys* is paraphyletic (Hanson & Bradley, 2008; Pine et al., 2012; Weksler, 2006), our results show the genera *Melanomys* and *Sigmodontomys* as reciprocally monophyletic clades with strong support each, at least based on their posterior probabilities (Figure 1). For the genus *Melanomys*, our analyses recovered with strong support the same species‐level haplogroups (*caliginosus, chrysomelas, columbianus, idoneus*) as Hanson and Bradley (2008). However, although we found a strongly supported *Melanomys* (only based on PP), the relationships for the major clades within the genus are not fully resolved; in fact, the only strongly supported relationship (in both PP and BS) corresponds to a sister clade between *M. columbianus* and *M. idoneus*. Noteworthy, a polytomy is found for three other clades, *caliginosus*, *chrysomelas*, and a group of sequences from Cordillera Central of Colombia (including also our sample JFD1322) that we provisionally name *Melanomys* sp.

## 4 DISCUSSION

Species identification efforts have drastically being influenced by the use of molecular data acting as a complement to taxonomic information obtained from morphology alone (Camargo & Sites, 2013; Carstens et al., 2013; Luo, Ling, Ho, & Zhu, 2018); however, the availability of molecular datasets (genes or genomes) is still restricted to particular biotic groups, while many others—in which high diversity occurs—lack proper molecular information, hindering the ability of addressing evolutionary questions, biogeographic hypothesis, and accurate species delimitation and identification (Helmy, Awad, & Mosa, 2016; Noreña, González Muñoz, Mosquera‐Rendón, Botero, & Cristancho, 2018). Traditional barcoding approaches have proven useful for species identification (Barco, Raupach, Laakmann, Neumann, & Knebelsberger, 2016; Hebert, Ratnasingham, et al., 2003), but they are not suitable for certain taxa lacking reference genomes or specific primers for PCR amplification when universal primers do not work as expected (Ford et al., 2009; Moulton, Song, & Whiting, 2010; Pino‐Bodas, Martín, Burgaz, & Lumbsch, 2013). We showed that Oxford Nanopore Sequencing stands as an alternative to facilitate the sequencing of organellar genomes (via genome skimming) for metazoan research where mitochondrial genomes could be easily generated PCR‐free. Also, given accessibility and cost–benefit of the technology, this could be a useful tool to reduce the gap in DNA sequence knowledge for nonmodel organisms. However, although promising, several methodological aspects ranging from library preparation, data analyses, and systematic revisions of understudied groups need to be addressed to fully implement a methodological approach for organelle sequencing and DNA‐based species identification as the one proposed here.

### 4.1 DNA sequencing of mitochondrion using ONT‐only data

Comparison of the two DNA extraction methods showed that HMW extraction outperforms the extraction kit in terms of both the amount of DNA obtained (~65 µg compared to ~4 µg) and DNA integrity (see Figure S1). However, the lack of replicates in our treatments (HMW vs. extraction kit) and the absence of using multiple extraction kits prevent us from making a generalization about the benefit of using HMW methods for DNA extraction. Nonetheless, we could observe that an appropriate selection of DNA extraction method prior to library preparation is crucial in terms of DNA yield and DNA integrity, which are two key elements required to take full advantage from the potential of long‐read sequencing technologies (Mayjonade et al., 2016).

Additionally, it is important to note that data analyses to generate mitochondrial genomes (via genome skimming) exclusively using ONT data are not entirely a straightforward process. Other studies have shown complete mtDNA assemblies combining ONT datasets and high‐throughput short‐read datasets from Illumina to improve overall consensus quality (Chandler et al., 2017; Torres et al., 2018). For instance, the complete mitogenome assembly of a clinical‐interest nematode, *Nippostrogylus brasiliensis*, was improved using dual datasets (Chandler et al., 2017). The need of Illumina reads for correcting ONT's sequencing errors was a relevant strategy because the authors observed discrepancies when mapping ONT raw reads to the assembly consensus, some of which were attributable to DNA methylation and epigenetic modifications (Chandler et al., 2017). We experienced similar complications when reconstructing the mitochondrial assembly using a simple de novo assembly pipeline from mtDNA reads (Minimap/Miniasm‐RACON). During the annotation process, we observed multiple instances of truncated ORFs for several coding genes due to premature stop codons. Although such misassemblies were particularly observed in polynucleotide regions, they were also observed in other sequence contexts. This could be due to the presence of modified nucleotides (i.e., different types of DNA methylation), which affects basecalling, and consequently misasigning bases, as has occurred in other studies (Stoiber et al., 2017). To solve this problem, we used the correction features included in Nanopolish (methylation aware correction and homopolymer correction), and although they fixed some frameshifts and substitutions, those approaches were not enough to obtain an assembly free of error. Consequently, it was necessary to combine different assembly routines in basecalling and correction steps (number of runs of RACON or Nanopolish) to produce four different alignments that allowed us to generate a “prioritized consensus” sequence of the mitochondrial genome. By “prioritized consensus,” we mean that in the presence of polymorphisms, we preferred the one preserving the reading frame of the protein‐coding sequence or the RNA stability, depending on the region where it occurred. Based on our proposed method—and despite the previously mentioned basecalling issues—it was possible to obtain *“*de novo” a complete mitochondrial genome using ONT‐only sequencing data.

Despite our results, the mtDNA content was low when compared to the overall amount of DNA isolated from the working tissue. In order to increase sequenced mtDNA yield (0.53% of total sequenced bases), further adjustments in DNA extraction should be developed to increase mtDNA fraction. In previous experiments, we attempted a strategy focused on isolation of enriched mitochondrial fractions from liver tissue (Frezza, Cipolat, & Scorrano, 2007) followed by a DNA extraction, but we did not obtain any improvement in mtDNA:nDNA ratio compared with a total DNA extraction (data not shown); moreover, DNA fragment size was considerably lower, possibly due to the mechanical stress induced by additional sample manipulation (e.g., pipetting, vortexing, centrifugations). The implementation of protocols that have been shown to provide an enriched mitochondrial fraction (eventually allowing to multiplex several mtDNA samples per flow cell) such as the Abcam mitochondrial isolation kit (Song et al., 2017; Sun et al., 2016), sucrose gradients (Stockburger et al., 2015), or Nycodenz gradients (Gaudioso, Garcia‐Rozas, Casarejos, Pastor, & Rodriguez‐Navarro, 2019) should be welcome; nonetheless, regardless of the protocol implemented, it is important to consider one that reduces the mechanical stress of the samples (preserving DNA integrity) and avoids PCR steps, if we are to maximize the value of this long‐read sequencing technology and its potential implementation under field conditions.

### 4.2 Species identification using ONT data

We explored the use of sequencing reads prior any assembly strategy to apply a species identification method for metazoan organisms based on mitochondrial information. The approach used here could successfully identify the organism of study using mitochondrial reads even in its uncorrected form (i.e., raw reads without any polishing, error rate correction, or any assembly). The selection of mtDNA for this task was not trivial, given its substitution rate and relative rapid coalescent times, this marker has been traditionally used to discriminate taxa at the species level (Hudson & Turelli, 2003), and consequently, there is a wealth of mitochondrial data in sequence repositories that can be used for comparison purposes. Prefiltering mitochondrial sequencing reads using low stringency BLAST parameters allowed us to recover reads from a variety of metazoan groups other than our particular rodent species. This can be viewed as advantageous for two reasons: Firstly, the identification is not researcher‐biased towards a particular taxonomic group (i.e., cricetids only, in this case) providing improved sensitivity as observed in the complete analysis (Figure 5a); and secondly, hits from different orders were recovered with considerable high identities (above 90%), an event that seems to provide information on the habitat of the sampled organism (e.g., species of the local community). Additionally, for the molecular species identification, we observed that any a priori information to delimit the database reduces substantially the search time, allowing to obtain an identification in a matter of minutes.

The present work contributes to the development of a recent field of research that aims to provide a DNA‐based species identification in real‐time and under field conditions. There are currently several methods (laboratory and computational) for bacterial and viral identification based on environmental samples (Batovska, Lynch, Rodoni, Sawbridge, & Cogan, 2017; Schmidt et al., 2017), 16S rDNA amplification with no prior isolation of taxa (Benítez‐Páez et al., 2016), and whole‐sample sequencing including analytical pipelines for real‐time identification such as WIMP (Juul et al., 2015). In plants, field‐based setups have been evaluated also in a DNA barcode‐free protocol (using total DNA samples) where the identification has been performed using BLAST‐based strategies, similar to ours, but in contrast, the identified plant species had plenty of DNA sequences in public databases (i.e., species from the genus *Arabidopsis*), which is not the case for the neotropical rodent scenario (Parker, Helmstetter, Devey, Wilkinson, & Papadopulos, 2017).

Recently, a genome skimming strategy using MinION data was applied to the illegal trade case of shark fins in India. mtDNA and nuclear loci were successfully recovered (coverage of 20×) and used for identifying the original specimen. The tissue was associated with a threatened shark species included in CITES (Johri et al., 2019). In our case, whole mitochondrion was recovered with 78× average coverage, but specimen identification could be performed with the available loci in databases (*COI* and *CYTB* mostly.).

Important advances have been also made in field‐based vertebrate species identification, although in a DNA barcoding context, requiring PCR and facing its associated problems (Pomerantz et al., 2018). Our method is particularly inclined towards the identification of metazoan species using mtDNA sequences and implementing a PCR‐free genome skimming strategy. By prefiltering the mitochondrial DNA sequences (from the total sequenced genomic DNA), our method saves computing time while it also improves the ability of the method to positively identify the species since multiple mitochondrial loci are available in public databases for many metazoan specimens (opposed to the comparatively low availability of nuclear sequences for most nonmodel organisms). Finally, automated species identification methods (such as the one proposed here) entirely rely on the identification associated to the sequences present in repositories (e.g., BOLD, GenBank), and consequently, erroneous identifications of sequences in such repositories or lack of systematic revisions in the group of interest (see below) may misguide the identification of “unknown samples.”

### 4.3 Systematics of the genus *Melanomys*

The genus *Melanomys* has been recognized as monophyletic based on parsimony analyses of morphological characters (Pine et al., 2012), and many diagnostic traits support such arrangement (see Weksler & Lóss, 2015); nonetheless, other molecular‐based phylogenetic reconstructions have rendered the genus *Melanomys* as polyphyletic (Hanson & Bradley, 2008; Pine et al., 2012), and consequently, new analyses were needed in order to add evidence to either hypothesis. Our analyses are largely based on the same sequences used by Hanson and Bradley (2008); however, we included an expanded taxon sampling, which results in a strongly supported monophyletic *Melanomys* based on posterior probability values. Incomplete taxon sampling has been shown to increase error in phylogenetic reconstructions (Heath, Zwickl, Kim, & Hillis, 2008), and therefore, the addition of new lineages could explain our results; however, bootstrap values are still low in support of the monophyletic *Melanomys,* and new analyses (including the extended taxon sampling and adding new markers other than the mitochondrial) should still be welcome to test our results.

As currently understood, *Melanomys* is a polytypic genus with 6 recognized species, 4 of which are included in our phylogenetic analyses (Figure 1). The species *M. caliginosus* has the broadest distribution within the genus, ranging along the Andean cordillera from southern Ecuador to northern Colombia, but it also occurs on the Pacific lowlands of both countries; in fact, its holotype was collected in Esmeraldas (see Allen, 1913), an Ecuadorian province in the lowlands of Choco. Although populations from the north of Cordillera Central of Colombia have traditionally been referred to the species *M. caliginosus* (Weksler & Lóss, 2015), based on our results we cannot assign with confidence any name to the haplotypes from the northern Andes based on the following reasons: (a) Our *CYTB* topology includes material that on the basis of geography itself is considered topotypical for the name *caliginosus* (locality 8 in Figure 1); nonetheless, such haplogroups do not form a monophyletic group with haplotypes from the northern Andes of Colombia; (b) as in the previous numeral, none of the other species included in our sampling (*chrysomelas, columbianus, idoneus*) forms a clade with haplotypes from northern Colombia; (c) based mainly on distribution, the species *M. robustulus* and *M. zunigae* (for which sequence data are unavailable) are unlikely associated with populations of the northern Andes given their broadly allopatric distributions in the western Amazon and the western coast of Central Peru, respectively; (d) uncorrected *p*‐distances between our haplogroup from the north of Cordillera Central and other haplogroups (i.e., species) of the genus range between 6.5% and 7.6% (Table S2), values that fall within the interspecific variation recorded in several other species of sigmodontine rodents (D'Elía, Hanson, Mauldin, Teta, & Pardiñas, 2015; D'Elía, Pardiñas, Jayat, & Salazar‐Bravo, 2008); (e) although our phylogenetic reconstruction supports the hypothesis of other authors in recognizing the species *M. caliginosus* as clearly polytypic (Weksler & Lóss, 2015), and despite the fact that other names—based on geographic proximity to type locality—could apply to our specimens (e.g., *obscurior* Thomas, 1894), we lack the geographic sampling along the Andes of Colombia and Ecuador to support any unambiguous identification. It is clear that the genus *Melanomys* is in urgent need of a systematic revision, and until such work is developed, we defer to apply any name to the populations included in the present study.

## CONFLICT OF INTERESTS

None declared.

## AUTHOR CONTRIBUTIONS

NDF and JFD conceived and designed the study. JFD performed fieldwork, specimen sampling, and morphological identification. NDF carried out molecular experiments, ONT sequencing runs, and computational data analysis. NDF and JFD wrote the manuscript.

### Open Research Badges

This article has earned an Open Data Badge for making publicly available the digitally‐shareable data necessary to reproduce the reported results. The data is available at Bioproject http://www.ncbi.nlm.nih.gov/bioproject/492505; Sequences https://www.ncbi.nlm.nih.gov/nuccore/MH939287.1, https://www.ncbi.nlm.nih.gov/nuccore/MH939280.1, https://www.ncbi.nlm.nih.gov/nuccore/MH939281.1, https://www.ncbi.nlm.nih.gov/nuccore/MH939282.1, https://www.ncbi.nlm.nih.gov/nuccore/MH939283.1, https://www.ncbi.nlm.nih.gov/nuccore/MH939284.1, https://www.ncbi.nlm.nih.gov/nuccore/MH939285.1, https://www.ncbi.nlm.nih.gov/nuccore/MH939286.1.

## Supporting information

AppendixS1

## Data Availability

Data are available from NCBI BioProject under BioProject ID: PRJNA492505, BioSample accession: SAMN10103913, and SRA accession: PRJNA492505. Final mitochondrial DNA assembly data are available under GenBank accession MH939287. Partial mitochondrial gene sequence data by Sanger method are available under GenBank accessions MH939280–MH939286.
