# Supplementary material for: Rapid mitochondrial genome sequencing based on Oxford Nanopore Sequencing and a proxy for vertebrate species identification
Source: Ecol Evol. 2020 Mar 11;10(7):3544–60. doi: 10.1002/ece3.6151 (PMC7141017; doi:10.1002/ece3.6151)
Supplement: Supplementary file 1 — AppendixS1 [file ECE3-10-3544-s001.docx]

**Supporting information for:**

***Rapid mitochondrial genome sequencing based on Oxford Nanopore Sequencing and a proxy for Vertebrate Species Identification using MinION device***

Authors:

Nicolás D. Franco-Sierra, Juan F. Díaz-Nieto

**Figure S1**. Low-agarose gel electrophoresis (0.5%). GeneRuler 1 kb was used as DNA ladder (lane 1). DNA obtained from the HMW protocol shows little smearing pattern and high molecular weights, over 10 kbp (lanes 2 and 3). DNA obtained from extraction using commercial GenElute kit shows a strong smearing pattern (lanes 4 and 5).


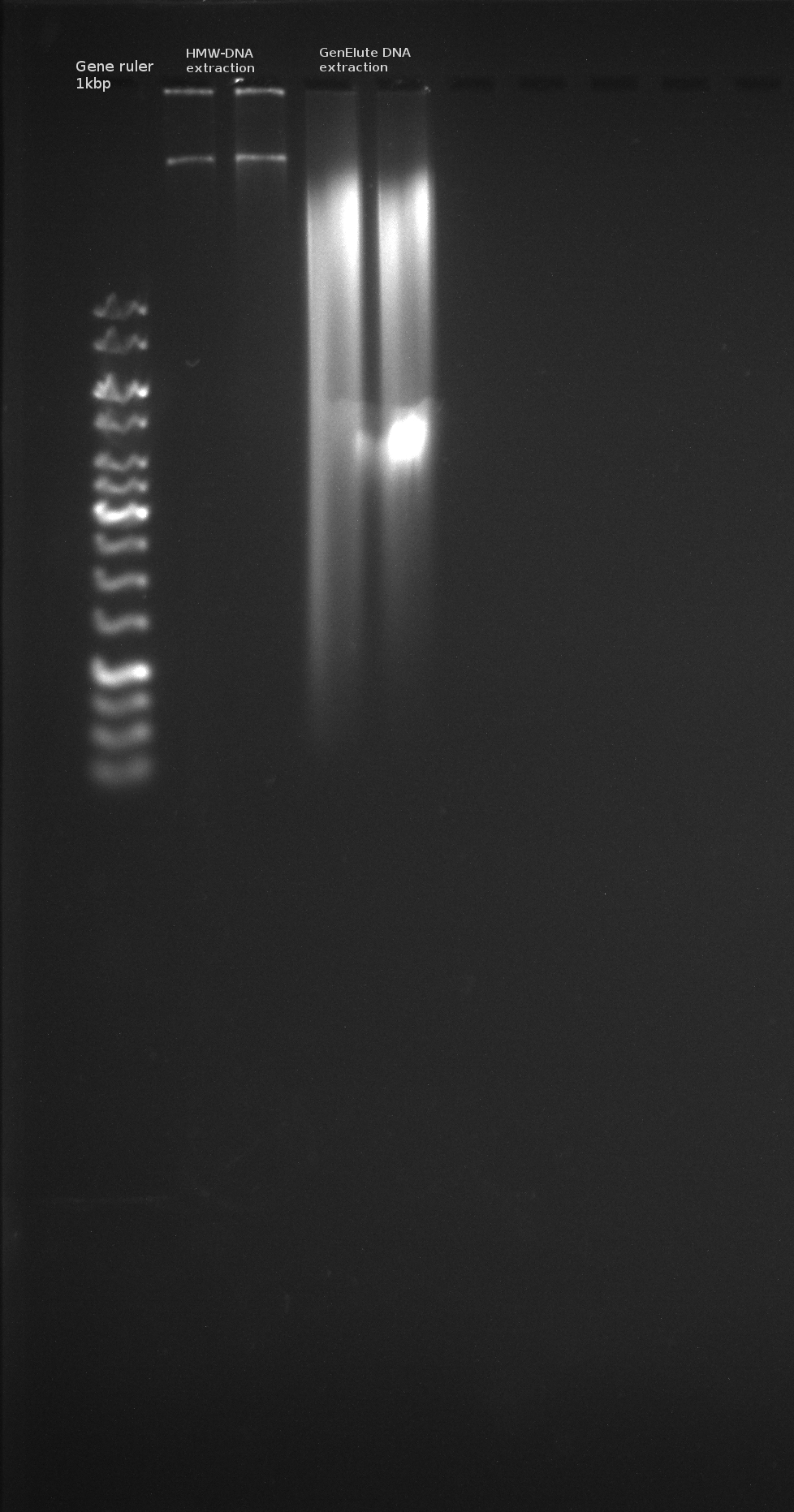


**Table S1.** Sequences of the mitochondrial cytochrome b oxidase gene (ingroup and outgroup) obtained for our phylogenetic analyses. Identification of the sequences follow Hanson & Bradley (2008) and Weksler & Lóss (2015), except for *Melanomys* sp. (see text).

| **Species** | **Catalogue number** | **Genbank accession number** | **Sequence length (bp)** | **Locality** | **Latitude** | **Longitude** |
| --- | --- | --- | --- | --- | --- | --- |
| *Melanomys chrysomelas* | TTU-M100313 | EU074633.1 | 1143 | Nicaragua: RAAN, Rosa Grande | 13.64061 | -85.01846 |
|  | TTU-M100309 | EU340018.1 | 1143 | Nicaragua: Atlántico Norte, Rosa Grande, Siuna | 13.64061 | -85.01846 |
|  | TTU-M100324 | EU340017.1 | 1143 | Nicaragua: Atlántico Norte, Rosa Grande, El Balsamo | 13.66164 | -84.96485 |
|  | RMT4658 | EU665204.1 | 1143 | Costa Rica: Prov. Heredia, La Selva Biological Station, La Guaria | 10.19 | -84.13 |
|  | USNM:MAMM:464387 | KY754031.1 | 1129 | Panamá: Bocas Del Toro, Isla Bastimentos, Old Point | 9.3 | -82.11 |
| *Melanomys* sp. | JFD01322 | MH939281.1 | 1122 | Colombia: Antioquia, Envigado,Transversal de la Montaña | 6.16 | -75.54 |
|  | DAG00150 | MH939282.1 | 1065 | Colombia: Antioquia, Caldas, Reserva del Alto de San Miguel | 6.02 | -75.62 |
|  | JFD00237 | MH939283.1 | 1000 | Colombia: Antioquia, Sabaneta, Vereda la Doctora | 6.13 | -75.61 |
|  | JFD00248 | MH939284.1 | 801 | Colombia: Antioquia, Bello, Vereda Sabana Larga | 6.32 | -75.62 |
|  | YXR00014 | MH939285.1 | 988 | Colombia: Antioquia, Sabaneta, La Romera | 6.12 | -75.60 |
|  | YXR00016 | MH939286.1 | 993 | Colombia: Antioquia, Sabaneta, La Romera | 6.12 | -75.60 |
| *Melanomys caliginosus* | TTU-M102975 | EU340021.1 | 1143 | Ecuador: Esmeraldas, Comuna San Fransisco de Bogotá | 1.07258 | -78.71150 |
|  | TTU-M135894 | EU340020.1 | 1143 | Ecuador: Esmeraldas, Comuna San Fransisco de Bogotá | 1.07258 | -78.71150 |
|  | TTU-M102727 | EU340019.1 | 1143 | Ecuador: El Oro, Zaruma, Cerro Urcu | -3.70000 | -79.61667 |
| *Melanomys idoneus* | TTU-M39150 | EU340024.1 | 1143 | Panamá: Darién, Cana | 7.78333 | -77.70000 |
|  | ROM116303 | EU340023.1 | 1143 | Panamá: Darién, Cana | 7.78333 | -77.70000 |
|  | LSUMZ:M-579 | KP778251.1 | 1143 | Panamá: Cana | 7.739569 | -77.693939 |
|  | LSUMZ:M-568 | KP778229.1 | 1143 | Panamá: Cana | 7.739569 | -77.693939 |
| *Melanomys columbianus* | MHNLS7698 | EU340022.1 | 1143 | Venezuela: Zulia, Misión Tukuko | 9.83333 | -72.86667 |
| *Sigmodontomys alfari* | USNM:MAMM:575662 | KY754155.1 | 1140 | Panamá: Bocas Del Toro, Ñuri | 8.91667 | -81.81667 |
|  | USNM449895 | EU074635.1 | 1143 | Panamá: Boca del Toro, Isla San Cristóbal | 9.25000 | -82.26667 |
|  | TTU-M103047 | EU340016.1 | 1143 | Ecuador: Esmeraldas, Estación Experimental 'La Chiquita' | 1.232 | -78.76603 |
| *Tanyuromys aphrastus* | KU161003 | JF693877.1 | 1107 | Costa Rica: Monteverde Cloud Forest Reserve | 10.30 | -84.79 |
| *Oryzomys palustris* | SCVA15 | EU074640.1 | 1143 | USA: Virginia, Norfolk Co | 36.69 | -76.45 |
|  | EVGL06 | EU074639.1 | 1143 | USA: Florida, Miami-Dade Co, Everglades National Park | 25.39 | -80.94 |

**Table S2.** Nucleotide distance matrix calculated from *CYTB* sequence alignment of ingroup species (including species of *Melanomys* and *Sigmodontomys*). Between group mean uncorrected *p*-distances are shown in lower left matrix, corrected between group mean distances using Tamura-Nei (Gamma) model are shown in the upper right matrix, and within group mean distances are shown along the diagonal.

|  | ***Melanomys chrysomelas*** | ***Melanomys* sp.** | ***Melanomys caliginosus*** | ***Melanomys idoneus*** | ***Melanomys columbianus*** | ***S. alfari*** |
| --- | --- | --- | --- | --- | --- | --- |
| ***Melanomys chrysomelas*** | 0.0081 | 0.0765 | 0.0780 | 0.0709 | 0.0706 | 0.0773 |
| ***Melanomys* sp.** | 0.0693 | 0.0040 | 0.0733 | 0.0713 | 0.0736 | 0.0716 |
| ***Melanomys caliginosus*** | 0.0707 | 0.0664 | 0.0181 | 0.0678 | 0.0746 | 0.0728 |
| ***Melanomys idoneus*** | 0.0650 | 0.0650 | 0.0617 | 0.0035 | 0.0456 | 0.0680 |
| ***Melanomys columbianus*** | 0.0649 | 0.0669 | 0.0677 | 0.0431 | - | 0.0732 |
| ***Sigmodontomys alfari*** | 0.0702 | 0.0650 | 0.0662 | 0.0618 | 0.0663 | 0.0275 |
